# Possible predictors of apneic-events during sleep: advanced snoring sound analysis

**DOI:** 10.1007/s11325-025-03568-w

**Published:** 2026-02-13

**Authors:** Malte Unkell, Maximilian Schmitt, Winfried Hohenhorst, Christoph Janott, Michael Herzog, Clemens Heiser

**Affiliations:** 1 Department of Otorhinolaryngology/Head and Neck Surgery, Klinikum Rechts der Isar, Technical University of Munich, Munich, Germany; 2 Department of Otorhinolaryngology/Head and Neck Surgery, Alfried Krupp Hospital, Essen, Germany; 3 Diametos GmbH, Potsdam, Germany; 4 Department of Otorhinolaryngology, Head and Neck Surgery, Carl-Thiem Klinikum Cottbus, Medical University Lausitz, Cottbus, Germany; 5 HNO-Zentrum Mangfall-Inn, Institute for Sleep Medicine, Bad Aibling, Germany; 6 Faculty of Medicine and Health Sciences, Translational Neurosciences, University of Antwerp, Antwerp, Belgium

**Keywords:** Snoring, Obstructive sleep apnea syndrome, Respiratory sounds, Apnea obstructive sleep

## Abstract

**Purpose:**

Snoring is the most common symptom in patients experiencing obstructive sleep apnea (OSA) and is reported by up to 94% of those affected. It has already been extensively proven that snoring of patients with OSA acoustically differs significantly from habitual snoring. For this study, snoring in patients with OSA is analyzed by its different components: pitch, formants (F1-F3), formant-ratio F2/F1, harmonic-noise-ratio (HNR), spectral energy ratio (SER). The objective of this study is to determine whether one or more acoustic parameters exhibit significant alterations or distinctive features immediately preceding an apneic episode, as compared to the patterns observed during simple snoring in individuals with obstructive sleep apnea (OSA). Identifying such preapneic indicators could facilitate the prediction of subsequent apnea events. With further technological advances and greater standardization, acoustical analysis could play a central role in the diagnosis and treatment of snoring and sleep apnea.

**Methods:**

Based on the “Munich-Passau Snore Sound Corpus” (MPSSC) as one of the world’s largest data sets for snoring sounds, retrospective data, consisting of 219 patients with OSA, were obtained using drug-induced sleep endoscopy (DISE). The mentioned components were retrieved and analyzed with the open source software „Praat“. Statistical testing was done with t-tests (Student and Welch) and Mann-Whitney-U-test.

**Results:**

For four parameters (F1, F2/F1, HNR, logSER) there are statistically significant differences between continuous/simple and preapneic snoring sounds.

**Conclusions:**

Preapneic snoring sounds seem to have different characteristics than continuous/simple snoring sounds. With further research, a simple acoustic differentiation for various diagnostic purposes could become possible.

## Introduction

 Snoring is a component of noise pollution. Those who are impacted experience reduced sleep duration or involuntary microsleep incidents during the daytime, as well as heart failure, diabetes, coronary heart disease, strokes, and depression [1–5].

Additionally, up to 94% of people with obstructive sleep apnea (OSA) report snoring, which is the most prevalent symptom of the condition [6, 7]. As screening tools, the frequency, intensity, and volume of snoring are thought to be fairly simple to determine and can already offer important insights into sleep apnea. While OSA severity cannot be predicted by frequency, its presence can [8]. Loudness and intensity are also helpful indicators of OSA and are correlated with the severity of the condition [9, 10].

The introductory section highlights the clinical relevance of the problem: snoring affects many individuals and may be associated with significant health risks. Since snoring is primarily defined by its acoustic characteristics, this study focuses on analyzing these sound properties in detail. Specifically, the investigation examines whether and to what extent preapneic snoring sounds in patients with OSA differ from their continuous/simple, habitual snoring sounds.

## Materials and methods

A sound can have different characteristics and can therefore be classified differently: The perceived pitch of a sound is determined by its fundamental frequency, while the arrangement and presence of harmonics shape the sound’s timbre [11]. However, it is also possible for a sound to be inharmonic, e.g. containing noise without a fundamental frequency. A mixture of (inharmonic) noise and harmonic sound is referred to as a sound event.

- **Pitch**: This is a description of the melody of a sound, i.e. the course of the fundamental frequency over time.
- **Formants**: The upper airways allow certain frequencies of the excitation signal generated by vocal fold vibrations to pass through during vowel production and attenuate others. These passing frequencies are referred to as “formants” and are commonly defined as F1-F4. F1 represents the lowest frequencies and F4 the highest. Formant analysis can be utilized to differentiate between primary snorers and those with OSA [11].
- **Formant ratio**: Based on the formants described, acoustic differences between obstructive and habitual snoring are already possible. The scientific literature shows that the opening of the mouth has a decisive influence on the first formant, while the movements of the tongue significantly influence the second formant [12]. In speech acoustics, the ratio of the second to the first formant (F2/F1 ratio) is therefore often used to characterize the articulation of vowels. In particular, a higher F2/F1 ratio typically indicates a front vowel with a higher tongue position, while a lower ratio indicates a back vowel with a lower tongue position [13]. This principle can be transferred to snoring.
- **Harmonic-Noise-Ratio (HNR)**: This parameter maps the ratio between periodic (harmonic) and aperiodic (noise/non-harmonic) components of a sound in dB. The aperiodic parts, for example, are caused during phonation as an acoustic by-product by the turbulent air flow at the glottis level [14, 15]. Analogous to the formants, this value also depends on different configurations of the vocal apparatus or the development of cavities in the upper airways [16].
- **Spectral Energy Ratio (SER)**: The spectral energy distribution indicates the ratio of energy components in snoring sounds above or below a predetermined cut-off frequency.

The “Munich-Passau Snore Sound Corpus” (MPSSC), one of the world’s largest data sets for snoring sounds, provides the basis for the present work [17]. It was developed to find out whether different anatomical locations of the origin of snoring sounds correlate with certain acoustic parameters.

The video and audio recordings that were created come from three different medical centers that perform DISE examinations as a diagnostic routine for selected patients:

- Klinikum rechts der Isar (Technical University of Munich): recordings of 38 patients between the years 2013–2014.
- Alfried Krupp Hospital Essen: recordings of 2090 patients between 2006 and 2015.
- University Hospital Halle/Saale: recordings of 46 patients between 2012 and 2015.

All patients underwent polysomnography (PSG) prior to the DISE examination and were diagnosed with OSA.

This retrospective dataset, comprising information from 219 patients after applying stringent exclusion criteria, was gathered through drug-induced sleep endoscopy (DISE) and stored in WAV file format.

The total number of snoring events selected based on previous exclusion criteria was 331 (Munich: 31, Essen: 266, Halle/Saale: 34) [17].

**Fig. 1:**
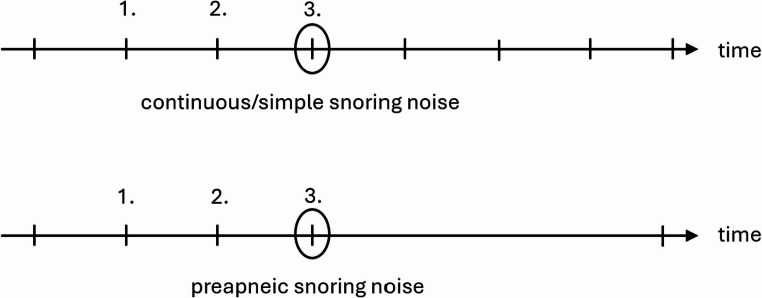
Visualization of measurement classification criteria for “continuous/simple” and “preapneic” snoring noise. Each line on the timeline represents a snoring noise. The circle around the 3rd line marks the measured snoring noise

**Fig. 2:**
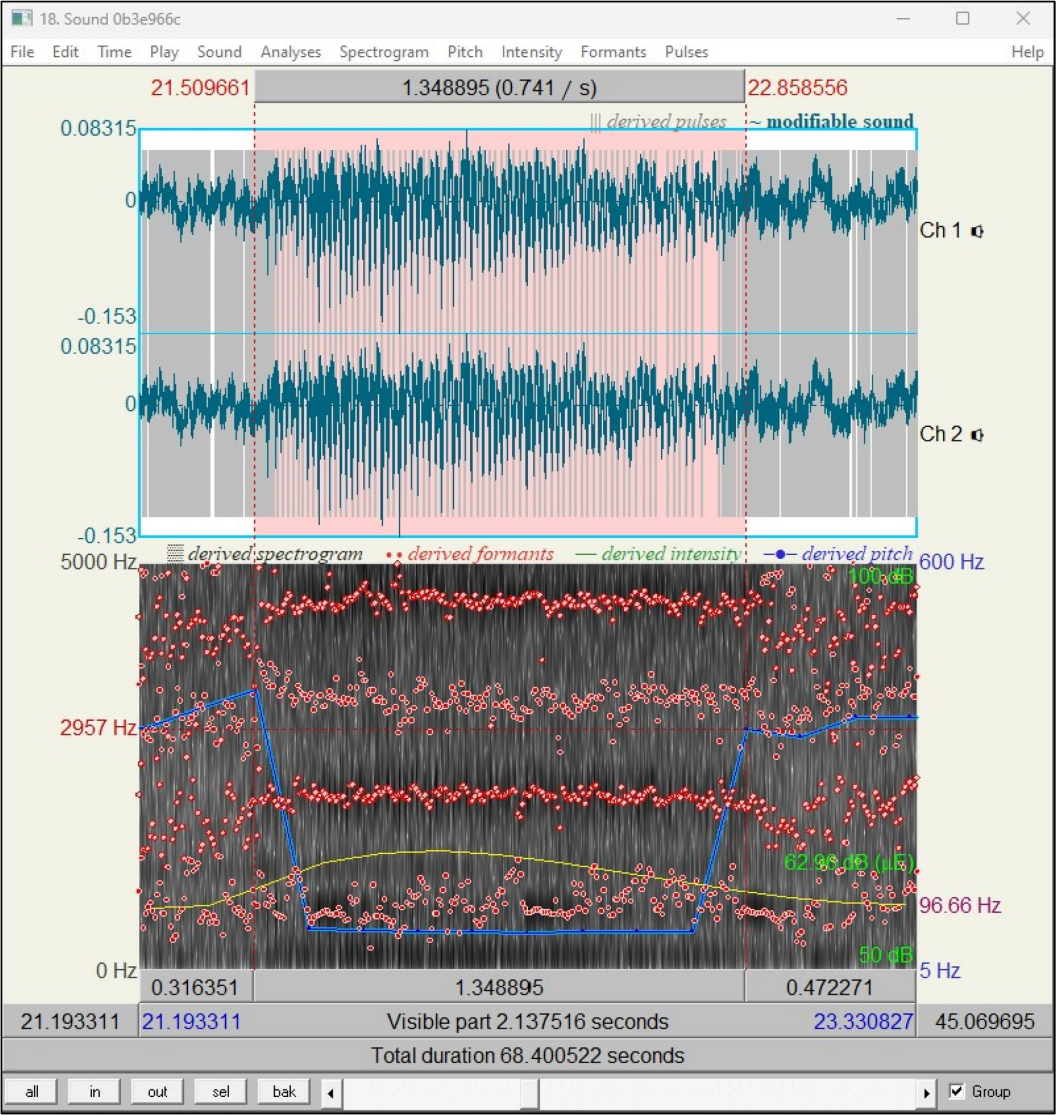
Praat tool interface with the marked snoring sound for analyzation

The individual videos of the respective DISE examinations from the corresponding centers were viewed and provided with time markers in an Excel table. These markers correspond either to snoring with subsequent apnea according to the American Academy of Sleep Medicine (AASM) definition or continuous/simple snoring according to the definition for this study (see Fig. 1). Simultaneous viewing of the videos and audio allowed the apneas to be visually confirmed and thus reliably identified. The audio track of the videos was extracted in mp3 or wav format using the “VLC Media Player” software. Subsequently, the relevant time points could be further processed using the previous temporal markers with the help of the open source software “Praat” (see Fig. 2) [18]. The snoring was marked and analyzed within the audio track. As demonstrated in Fig. 2, most of the described variables can be derived directly from the measurement of the program.

Only the SER was calculated through a simple computational method implemented with the programming language Python.

## Results

In the following, the data obtained from the MPSSC is statistically processed and evaluated.

To ensure comparability of the data, criteria were defined before the evaluation:

- a snoring episode is defined as at least three similar snoring events in a row.
- the third snoring sound of the snoring episode is measured.
- if the third and thus measured snoring sound of the episode is not followed by apnea but, for example, by another snoring sound, this sound is classified as “continuous” or “simple”.
- however, if an apnea follows the third and thus measured snoring sound of the episode, this sound is classified as “preapneic”.

Accordingly, an opening snore after an apnea, for example, which has completely different acoustic characteristics, cannot be included in the analysis. Furthermore, a single “snoring event” (a single intake of breath) after or before the next apnea is not considered preapneic snoring. According to the criteria mentioned, these sounds are not the third sound of the snoring episode. Also excluded are snoring noises during/after manual maneuvers for examination purposes (e.g. Esmarch handgrip). Also excluded are snoring noises that exhibit level jumps (e.g. velum to tongue snoring). Such phenomena could be visually verified using DISE recordings.

Table 1 shows the descriptive statistics for all previously explained parameters of the apnea and continuous/simple snoring groups in OSA patients.

To obtain a normally distributed SER for statistical testing, it first had to be converted to a logarithmic function. This is possible in the sense of “best practice in statistics” in order to compensate for the skewness (type and strength of the asymmetry) and to ensure a more valid interpretation of the results. This is only carried out for statistical analysis in order to investigate any differences between the groups using Welch’s t-test. The actual interpretation is based on the normal SER data [19]. The values for statistical testing can be found in Table 2. In order to search for differences within the individual variables, various t-tests (Student and Welch t-test) and the Mann-Whitney U-test were used. Significant differences (*p* < 0.05) were found in F1, F2/F1 ratio, HNR and logSER.

## Discussion

At the beginning of this section, it should be emphasized that this study does not compare the acoustics of primary and obstructive snoring. Rather, it examines the differences in patients diagnosed with OSA between their continuous/simple and preapneic snoring.

In the investigation of the formants, there are obvious contradictions with the current state of science, if the difference between primary and obstructive snorers is taken into account. However, comparing continuous/simple and obstructive snoring only in patients with OSA represents a new approach.

**Fig. 3:**
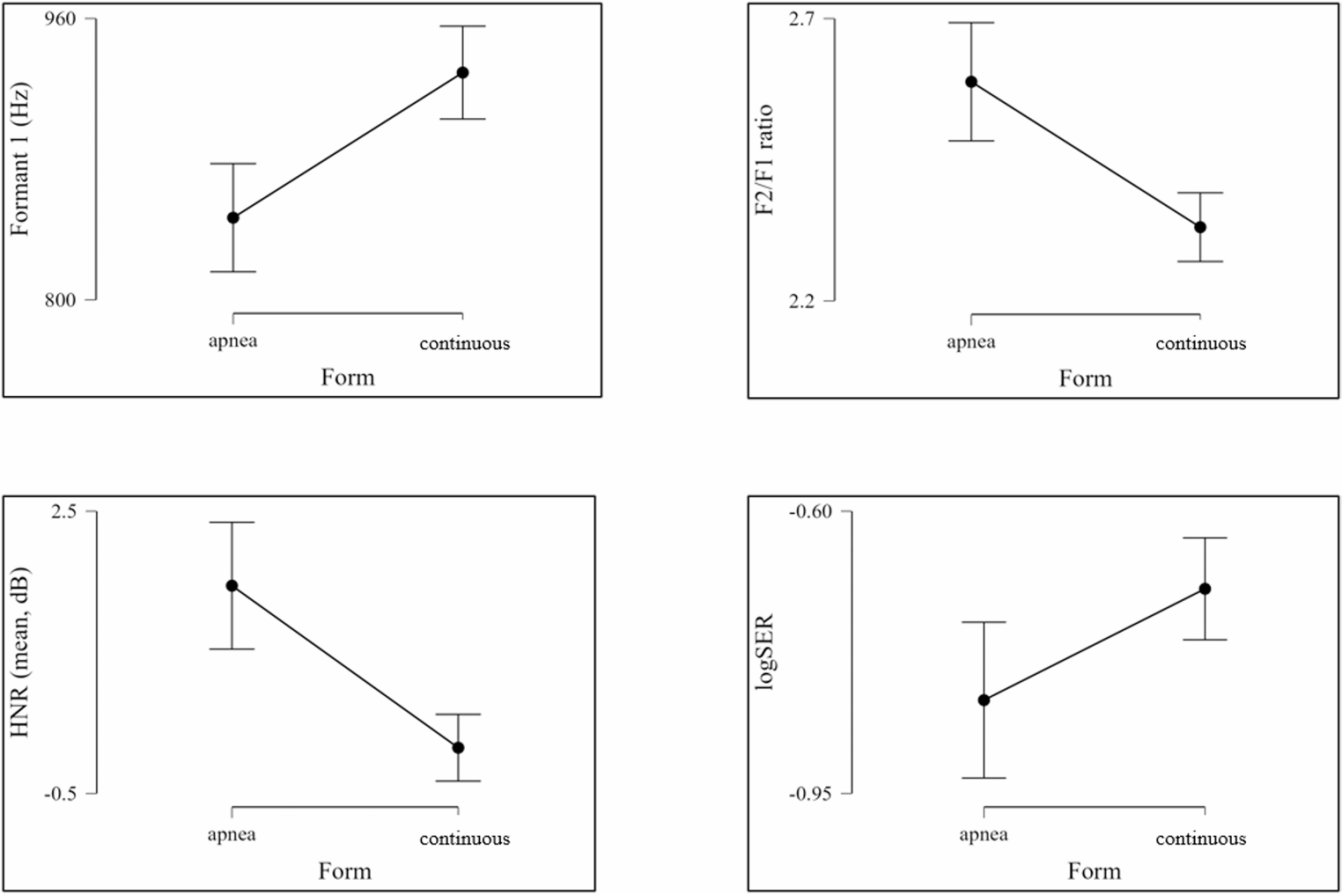
Visualization of differences between the two groups for the variables that show significant differences in statistical testing

The principle of formants can ultimately be transferred to snoring: Snoring as an excitatory signal, is not caused by vocal fold vibrations, but by vibrations of soft tissue at certain anatomical constrictions due to the air flow in the upper airways. There are no generally valid values. The length and formation of the cavities in the upper airways determine how pronounced a formant is. The formant analyses for testing possible differences between OSA and continuous/simple snoring are based on the assumption that primary snoring occurs primarily velopharyngeally and obstructive snoring primarily retrolingually or hypopharyngeally. It was found that the frequency of the first formant F1 is significantly higher in snorers with obstructive sleep apnea than in people who primarily snore and thus mainly occur retrolingually/hypopharyngeally [20]. Although a significant difference between continuous/simple and preapneic snoring was also found in preapneic snoring, especially in the F1 range, this was the opposite of the previous observation: in Fig. 3 and the statistics in Table 1 clearly show that the group of continuous/simple snoring sounds has higher preapneic frequencies than the obstructive snoring sounds. Thus, it should be noted that a difference of statistical small to moderate effect size definitely exists between the two groups but needs to be confirmed by further studies. This could mean that preapneic snoring has an individual acoustic profile in the first formant (F1) that differs significantly from the continuous/simple snoring sounds investigated so far.

**Table 1:** Descriptive statistics

	Formant 1		Formant 2		Formant 3		F2/F1 ratio		HNR		Pitch		SER		logSER	
	apnea	cont.	apnea	cont.	apnea	cont.	apnea	cont.	apnea	cont.	apnea	cont.	apnea	cont.	apnea	cont.
Mean	846.73	929.28	2046.89	2059.18 (Hz)	3218.38	3245.42	21,582	2.33 (Hz)	25,934	−0.01	176.95	206.48	0.48	0.50	−0.83	−0.70
	(Hz)	(Hz)	(Hz)		(Hz)	(Hz)	(Hz)		(dB)	(dB)	(Hz/s)	(Hz/s)				
Standard deviation	218.27	234.76	166.62	243.08	252.23	288.17	0.74	0.54 (Hz)	16,528	41,699	157.43	176.02	45,962	0.93	0.69	0.56
	(Hz)	(Hz)	(Hz)	(Hz)	(Hz)	(Hz)	(Hz)		(dB)	(dB)	(Hz/s)	(Hz/s)				
Minimum	369.80	422.60	1556.06	1410.62 (Hz)	2504.40	2518.22	14,977	13,516	−6.19	−6.60	45,782	45,662	0.00	0.01	−2.52	−2.10
	(Hz)	(Hz)	(Hz)		(Hz)	(Hz)	(Hz)	(Hz)	(dB)	(dB)	(Hz/s)	(Hz/s)				
Maximum	1435.87	1600.86	2451.54	2666.82 (Hz)	3803.52	3960.44	19,845	36,251	46,010	13.20	588.80	589.80	32,051	33,025	45,748	0.84
	(Hz)	(Hz)	(Hz)		(Hz)	(Hz)	(Hz)	(Hz)	(dB)	(dB)	(Hz/s)	(Hz/s)				

*HNR* harmonics to noise ratio, *SER* spectral energy ratio, *F* formant, *cont.* continuously, *Hz* Hertz, *dB* decibel, *Hz/s* Hertz per second, *SD* standard deviation, *Std.* standard

The results obtained with regard to the formants F2 and F3 supplement the current literature on acoustic analysis. This has so far indicated that the second (F2) and third formant (F3) do not have sufficient differentiating power to identify significant differences between primary and apneic snoring. This also applies to the comparison between continuous/simple and preapneic snoring. Previously, no significant correlation was found between the (higher frequency) formants F2 and F3 and the severity of obstructive sleep apnoea [11]. Consequently, these formants should not be used as reliable indicators to differentiate between these groups.

The ratio of the formants F2 to F1 can provide important information about the acoustic properties of the sound source and its position in the upper airways, particularly in the case of snoring sounds. The results obtained here show a significant difference between continuous/simple and preapneic snoring and thus allow a differentiation between them. This may be due to the onset of obstruction, which potentially leads to a higher resonance frequency of F2. Another conceivable explanation would be a preapneically growing involvement of the sublingual resonance chamber, which influences the resonance frequency relevant for F1, or a more extended resonance chamber without the onset of obstruction during continuous/simple snoring, which causes the lower resonance frequencies (F1). Another explanation could be that snoring associated with apnea is preferentially generated in the lower part of the pharynx, while non-obstructive snoring is primarily generated in the velopharyngeal region. The different localization of the sound source could influence the resulting resonance patterns, which in turn could have an impact on the ratio of the first two formants.

The stability of the fundamental frequency/pitch is an important sound characteristic and can serve as a distinguishing feature between primary and obstructive snoring. Quantitative frequency analyses have already shown that habitual snoring tends to occur in low-frequency spectra < 500 Hz, while snoring associated with OSA has intensity maxima > 500 (−1000) Hz on average [21, 22]. Furthermore, snoring in OSA contains reduced tonal components compared to primary snorers [11]. However, this finding is based on the premise that primary snoring takes place in the velopharyngeal region, while obstructive snoring takes place retrolingually or hypopharyngeally [23]. With regard to this variable, the non-significant result means that preapneic snoring sounds do not differ between continuous/simple and obstructive snoring in this component. This difference is also evident in Table 2 for the present study, but it should be noted here that these are patients with OSA who have been diagnosed with certainty. Similar to F1, the result obtained complements the current state of research, as a significant difference between the groups with primary and obstructive snoring is expected according to previous literature. However, the new approach in the consideration of continuous/simple and preapneic snoring in patients with OSA does not allow the same conclusions to be drawn as in the current study situation.

**Table 2:** t-test (Student and Welch) and Mann-Whitney U-test for independent samples

	Test	Statistics	*p*	Effect strength
Formant 1	Student	−3.95 (Hz)	< 0.001	−0.36
Formant 2	Welch	−0.67 (Hz)	0.502	−0.06
Formant 3	Student	−1.08 (Hz)	0.282	−0.10
F2/F1 ratio	Mann-Whitney	36145.00	< 0.001	0.21
HNR	Mann-Whitney	32281.00 (dB)	< 0.001	0.25
Pitch	Mann-Whitney	24806.00 (Hz/s)	0.086	−0.09
logSER	Welch	−2.36	0.019	−0.22

*Std.* standard, *HNR* harmonics to noise ratio, *F* formant, *SER* spectral to energy ratio, *Hz* Hertz, *dB* decibel, *Hz/s* Hertz per second

Note: In the Students T-test and Welch's test, the effect size is indicated with Cohen's d. In the Mann-Whitney test, the effect size is indicated with the rank-biserial correlation

For HNR in previous studies, there appear to be significant differences between the two groups studied for continuous/simple snoring, in which the OSA group has higher values with an increased aperiodic component, but measured inversely as NHR (noise to harmonics ratio) [24]. No absolute limit values are currently available for interpretation from the literature. For this reason, the values from research are observed and used as relative limits. In HNR, the significant differences with moderate effect size can serve as a possible important parameter to differentiate the described sounds in affected patients. One possible explanation could be the closure of the pharyngeal tissue following the preapneic sound: depending on the degree of already reduced/collapsed opening of the upper airway, the airflow becomes more turbulent and thus produces more harmonic sounds at the constrictions until complete closure and complete interruption of the airflow occurs. The mean value here means that the harmonic component in the apnea group for preapneic snoring sounds is significantly more pronounced than in the continuous/simple snorers. The aperiodic/additive component seems to play a significantly greater role here. The moderate statistical effect size emphasizes the HNR in the context of this work as the most important possible predictor in future practical application.

For logSER, there is no specific limit value, most studies worked with a value of 1 kHz. In this sense, primary snoring appears to have more low-frequency energy components compared to OSA snoring. So far, however, it has not been possible to differentiate directly between the groups to be examined on the basis of this acoustic characteristic [11, 23, 25]. The values researched so far from the literature are not absolute for this parameter either but are already suitable for orientation. The significant differences in the logSER are also potential markers that could make a subsequent apnea foreseeable. According to previous literature, both groups are in the range of primary snoring despite reliably diagnosed OSA with the measured values, as there are more low-frequency energy components and thus a ratio of < 1. The acoustic phenomena would therefore have to have originated on the soft palate according to logSER. This could be interpreted as a contradiction to the current state of science, however, it must again be differentiated that the present research carried out a study of continuous/simple to preapneic snoring noises in patients with OSA and thus refers to previous results, but as such was not yet investigated at the time. The significant difference shows that a distinction between the two groups is possible. This difference is possibly due to the fact that the preapneic smaller airway causes a concentration of energy in higher frequencies. In contrast, the continuous/simple snoring of patients with OSA potentially generates lower frequency vibrations due to a more constant airflow, analogous to the pathomechanism of primary snoring. Thus, the spectral energy would be more concentrated in lower frequency ranges. Another explanation for the difference could be the different resonance chambers: narrower or partially closed resonance chambers generate higher frequencies and lead to a spectral energy distribution in the higher ranges.

Similar studies have already investigated postapneic snoring in relation to habitual snoring, for example [20]. A comparable investigation utilized machine learning to analyze preapneic snoring sounds, thereby emphasizing the distinctive characteristics of preapneic snoring sound acoustics. This analysis also highlighted the potential for preapneic snoring sounds to serve as a predictor for impending respiratory events [26].

The present work must be checked for existing limitations and their potential influence on the results.

- No data on respiratory or airflow is available for the videos of the DISE examinations from the MPSSC. The apneas and hypopneas were therefore confirmed acoustically and visually. This could have an influence on the accuracy of the classification as apnea or hypopnea. Furthermore, the severity of the diagnosed OSA was not considered and it was not possible to correlate the snoring acoustics to different sleep stages. As the present study concentrated on acoustic characterization prior to respiratory events; possibly, the OSA severity of each patient diagnosed by PSG beforehand is not primarily relevant.
- Furthermore, the patients were not subdivided by constitutional characteristics such as gender, age, weight, etc. For this reason, no results and associated interpretations can be made regarding differences in the acoustic composition of snoring sounds regarding these subgroups.
- The utilization of DISE constitutes a limiting factor, as it does not accurately reflect natural sleep patterns. Moreover, no records of medication used during the DISE procedure are available. Snoring under sedation during DISE could have different acoustic characteristics than during natural sleep due to the probable change in its anatomical origin [27].
- The current state of research on the acoustics of snoring does not yet al.low universally valid statements to be made. Accordingly, there are currently no absolute limit values for any parameters, which means that only relative assessments of the researched results are possible.

The preapneic snoring sound in patients with OSA can be identified with its individual characteristics in comparison to previously investigated snoring sounds via various acoustic parameters.

Definite significant differences exist in the four values F1, F2/F1, HNR and logSER. No significant differences were measured in the remaining three parameters F2, F3 and pitch. If the results obtained can be confirmed in further research, there are acoustic features in preapneic snoring sounds that make it possible to predict upper airway obstruction in patients with OSA.

All previously obtained results extend previous research on the acoustic analysis of snoring sounds. In addition, this work is based on the largest data set of snoring sounds from a patient collective of over 200 participants currently. 

## Data Availability

The datasets generated during and/or analyzed during the current study are available from the corresponding author on reasonable request.

## Abbreviations

AASM: American Academy of Sleep Medicine
CPAP: continuous positive airway pressure
dB: decibel
df: degrees of freedom
DISE: drug induced sleep endoscopy
HNR: Harmonic-noise ratio
MPSSC: Munich-Passau Snore Sound Corpus
NHR: noise-harmonic ratio
OSA: obstructive sleep apnea
PSG: polysomnography
SER: spectral energy ratio
Std.: standard
